# A cross-sectional and spatial analysis of the prevalence of multimorbidity and its association with socioeconomic disadvantage in South Africa: A comparison between 2008 and 2012

**DOI:** 10.1016/j.socscimed.2016.06.055

**Published:** 2016-08

**Authors:** Amy Weimann, Dajun Dai, Tolu Oni

**Affiliations:** a University of Cape Town, Division of Public Health Medicine, School of Public Health and Family Medicine, Room 4.41, Entrance 5, Falmouth Building, Anzio Road, Observatory, 7925, Cape Town, South Africa; b Georgia State University, Department of Geosciences, Georgia State University, 24 Peachtree Center Avenue NE, Atlanta, GA 30303, United States

**Keywords:** South Africa, Multimorbidity, Socioeconomic disadvantage, Hypertension, Spatial analysis, Hot spots

## Abstract

This study utilised data from the National Income Dynamics Study, a longitudinal study with a sample of approximately 28 000 people, to investigate the cross-sectional and spatial distribution of multimorbidity and the association with socioeconomic disadvantage in South Africa for 2008 and 2012. Multimorbidity increased in prevalence from 2.73% to 2.84% in adults between 2008 and 2012 and was associated with age, socioeconomic deprivation, obesity and urban areas. Hypertension was found frequently coexisting with diabetes. Spatial analysis showed clusters (hot spots) of higher multimorbidity prevalence in parts of KwaZulu-Natal and the Eastern Cape, which compared with the socioeconomic disadvantage spatial pattern. Although these results were limited to a district level analysis, this study has provided a platform for future local level research and has provided insight into the socioeconomic determinants of disease multimorbidity within a developing country.

## Highlights

• In South Africa, multimorbidity increased in prevalence between 2008 and 2012.
• Hypertension is a significant and increasing health burden in South Africa.
• Hypertension is comorbid with tuberculosis, HIV, and most frequently with diabetes.
• Multimorbidity hot spots were found in the eastern parts of the country.
• Deprivation, urban geography, female sex and obesity associate with multimorbidity.

## 1 Introduction

South Africa is one of the most developed and urbanised sub-Saharan African countries with approximately two thirds of its national population inhabiting urban areas (McGranahan and Martine, 2012). South Africa provides a remarkable backdrop for health geography research. The notorious Apartheid legacy of South Africa, which divided the population along racial lines and deprived the majority of South Africans of basic human rights, subsequently resulted in many social and economic injustices. The removal of the Apartheid laws in 1994 resulted in high levels of urbanisation and a rise in unemployment rates, which further entrenched inequality (Chopra and Sanders, 2004). Today, many disparities currently exist in South Africa, not only along socioeconomic lines but also spatially, such as between cities and the traditional homelands, and between districts and provinces (McIntyre et al., 2002, Noble and Wright, 2013).

Omran’s epidemiological transition model (Omran, 1971) provides a useful theoretical framework for national health research and suggests that a country’s health and disease pattern will gradually progress through a series of stages, in response to various social, economic and demographic factors. This model suggests that countries will evolve through an era dominated by infectious diseases before experiencing an era of chronic non-communicable diseases (NCDs), mostly caused by the adoption of unhealthy, sedentary lifestyles that include reduced exercise, poor nutritional intake, alcohol consumption and smoking tobacco products (Chopra and Sanders, 2004, Omran, 1971). However, some countries and sub-national populations do not experience a distinct progression through these stages and may in fact experience a blurry transition where infectious and non-communicable diseases exist together within a population and the poor may be disproportionately affected by disease. Thus, Frenk et al. (1989) presented the protracted-polarised model as a modification to the epidemiological transition model in order to represent this indistinct transition in epidemiology that is particularly relevant to developing countries of the global South.

The history of social, economic and demographic shifts in South Africa have all contributed towards a transition in health that supports the protracted-polarised model of epidemiological transition (Chopra and Sanders, 2004). South Africa is characterised by a quadruple burden of disease, which includes the human immunodeficiency virus (HIV) and tuberculosis (TB); NCDs; perinatal and maternal; and injury-related disorders (Bradshaw et al., 2003). In addition, the national population is increasingly aging and the widespread use of antiretroviral therapy is resulting in an aging population of HIV-infected persons and an accompanying rise in coexisting chronic health conditions in individuals (Tseng et al., 2014).

Multimorbidity, which is defined as the presence of two or more chronic health conditions existing simultaneously in an individual, brings about a decline in quality of life for patients as well as increased expenses and complications for treatment plans and medical care (Barnett et al., 2012, Marengoni et al., 2011). Multimorbidity has usually been associated with age, particularly with adults older than 65 years and is increasingly common in patients due to factors such as aging populations and a rise in age-related chronic health conditions (Uijen and van de Lisdonk, 2008, Van den Akker et al., 1998). However, recently it has been increasingly reported in people younger than 65 years, especially in adults who are socioeconomically deprived (Alaba and Chola, 2013, Barnett et al., 2012, Uijen and van de Lisdonk, 2008).

In South Africa, multimorbidity is alleged to be present in the population, however the prevalence and social determinants of multimorbidity remain under-researched (Alaba and Chola, 2013). Multimorbidity has been reported to be higher among females and in approximately 4% of the adult population; however it was suggested that this prevalence was likely underestimated (Alaba and Chola, 2013). High blood pressure is a common contributor to multimorbidity in South Africa (Alaba and Chola, 2013, Ataguba, 2013) and is frequently found to coexist with diabetes (Oni et al., 2015). Data from the 1998 South African Demographic and Health Survey (DHS), as reported in the Poverty and Chronic Diseases Report, suggest that areas associated with low socioeconomic status experience a significant burden of premature mortality that can be linked to various diseases (Bradshaw and Steyn, 2001).

The presence of socioeconomic factors such as crowded living conditions, poor nutrition, limited financial resources, and poor housing and sanitation, is likely to increase the risk of TB infection and exacerbate the transmission of the disease in communities, particularly where there is a high HIV prevalence (Creswell et al., 2011, Lönnroth et al., 2010, Rasanathan et al., 2011). Yet its influence on multimorbidity remains largely unknown. HIV was estimated to be prevalent in 12.2% of the population in 2012, according to the South African National HIV Prevalence, Incidence and Behaviour Survey (Shisana et al., 2014) and is the strongest known driver of the TB epidemic in South Africa, which was one of six countries with the highest number of new TB cases in 2013 (410,000–520,000 incident cases) (Creswell et al., 2011, World Health Organization, 2014). In addition, poorer socioeconomic groups have been associated with a higher prevalence of NCD risk factors, including alcohol consumption, increased salt consumption, and obesity which affects 40% of South African adult females (Bradshaw and Steyn, 2001).

Although hypertension has been associated with factors such as alcohol consumption, smoking, high body mass index and inadequate exercise, research has suggested that the degree of association between socioeconomic status and hypertension varies between males and females (Cois and Ehrlich, 2014). Data on hypertension prevalence for the country are available from the 1998 South African DHS, which show a prevalence of 21% for both males and females using the 140/90 mmHg threshold (Steyn, 2006). Cois and Ehrlich (2014) suggest this may have increased by approximately 22% and 28% in males and females, respectively, between 1998 and 2008.

Regarding diabetes, 6.5% of South African adults (20–79 years) were estimated to have diabetes in 2011 (Whiting et al., 2011). South Africa has very few prevalence statistics for diabetes, yet studies have shown an association with age and have revealed prevalence disparities between ethnic groups, with the Asian/Indian population at higher risk of developing type 2 diabetes due to a higher risk of developing insulin resistance compared to other ethnic groups (Bajaj and Banerji, 2004, Bradshaw et al., 2003). It is a common perception that diabetes is associated with urbanisation due to exposure to more sedentary lifestyles (Green et al., 2003, Shaw et al., 2010).

In South Africa, there is limited research investigating the coexistence of many of these diseases, as well as the association between multimorbidity and socioeconomic disadvantage (Ataguba, 2013). Therefore, this study aims to contribute to efforts in addressing the paucity of information on multimorbidity in South Africa by studying the epidemiology of selected chronic infectious and non-communicable diseases and multimorbidity, and the association with socioeconomic disadvantage in South Africa.

## 2 Materials and methods

This study utilised data from wave 1 (Southern Africa Labour and Development Research Unit, 2014a) and wave 3 (Southern Africa Labour and Development Research Unit, 2014b) of the National Income Dynamics Study (NIDS) to estimate the prevalence of HIV, TB and NCD multimorbidity for the adult sub-sample, focusing on hypertension and diabetes, the most prevalent NCDs in this setting, and to determine the changes in reported HIV, TB and NCD multimorbidity over time. In addition, this study compared the cross-sectional and spatial association between socioeconomic disadvantage and multimorbidity in respondents who completed wave 1 and wave 3 of the NIDS. Wave 2 was omitted from this study as response rates for key health-related questions determining if the respondent had diabetes or hypertension were considerably lower in wave 2. Further investigation of wave 2 data is necessary before its inclusion in research.

The NIDS is a panel study conducted by the Southern Africa Labour and Development Research unit (SALDRU) that seeks to provide representative socioeconomic, behavioural and anthropometric data for South Africa. This longitudinal study began in 2008 with a nationally representative sample of over 28,000 individuals, including adults and children from 7300 households (Leibbrandt et al., 2009). There have been three waves of data collection, in 2008, 2010 and 2012. The 2008 NIDS base wave used a stratified, two-stage cluster sample design. Each NIDS wave is regarded as an independent cross-section in order to maintain representation of the national population.

The study population under analysis was the adult sub-sample from wave 1 (2008) and wave 3 (2012) of the NIDS. It is important to note that the NIDS study primarily relies on self-reporting of health conditions, however blood pressure, height and weight measurements are taken as part of the survey.

### 2.1 Measures

The primary outcome variables in this study were multimorbidity and hypertension, which was identified as the most prevalent of the selected chronic diseases and most likely to contribute to multimorbidity. Respondents were classified as hypertensive if they acknowledged having ever been diagnosed with high blood pressure by a health care professional (self-reported measure) or if respondents had an average systolic blood pressure reading >140 mmHg and/or an average diastolic pressure reading >90 mmHg during the NIDS assessment (Steyn, 2006, Whitworth, 2003). Measurements were retained if the diastolic blood pressure was >30 mmHg and if the systolic blood pressure reading was between 80 and 240 mmHg.

Descriptive variables included age (originally treated as a continuous variable but later categorised), socioeconomic disadvantage (using a multidimensional poverty index), gender, racial groups as classified by NIDS, and the urban and rural geographies. According to Statistics South Africa (2007, p. 21–22), an urban area contains “formal cities and towns characterised by higher population densities, high levels of economic activities and high levels of infrastructure”, while a rural area comprises “farms and traditional areas characterised by low population densities, low levels of economic activity and low levels of infrastructure“. Risk factor variables were explored and four were selected for inclusion in this study, namely alcohol drinking status, smoking status, exercise and obesity. Respondents were classified as obese if their BMI score (kg/m^2^) was ≥30 (World Health Organization, 1995).

A multidimensional poverty index (MDPI) was used to provide a proxy indictor for socioeconomic disadvantage. The MDPI was based on the Acute Multidimensional Poverty Index for Developing Countries, as developed by the Oxford Poverty and Human Development Initiative for the United Nations Development Programme (Alkire and Santos, 2010). The index contains 10 indicators distributed under three dimensions: the education and health dimensions each contain two indicators, and the standards of living dimension contains six indicators. Each of the dimensions and the indicators within each dimension are equally weighted. A final score was calculated for each respondent and individuals were classified based on the following classification score by Alkire et al. (2014): if respondents were deprived in <20% of the weighted indicators, they were classified as ‘Not Deprived’; if deprived in 20%–33.3% of indicators, they are ‘Vulnerable to Poverty’; if deprived in 34%–49.9% of indicators, they are ‘Deprived’; and if deprived in ≥50% of indicators, they are in ‘Severe Poverty’. For the spatial analysis, respondents were classified as socioeconomically disadvantaged if they were classified as either ‘Deprived’ or in ‘Severe Poverty’.

In this study, data from wave 1 was used to calculate the MDPI so that a baseline socioeconomic score could be assigned to each individual. These scores were aggregated to provide information for spatial and temporal pattern analysis.

### 2.2 Statistical approach

All descriptive and statistical data analyses were performed using Stata (StataCorp, 2013) software (version 13.1). Survey design and post stratification weights were applied to the data. Descriptive statistics of the sample were presented using median, interquartile range and full range for continuous data and proportions and frequencies for categorical data. Exploratory bivariate analysis was performed using tabulations and chi-squared tests for categorical variables and logistic regression for assessing the association between selected dichotomous outcome variables and continuous data. Age categories were assigned to the age variable so that the distribution of health and risk factors may be explored by age group. Confidence intervals were set at 95% and values were considered statistically significant if p = <0.05. All variables that showed a statistically significant association with the outcome variable or that were potential confounding variables were included in the multivariable analysis.

Multivariable analysis was performed using two logistic regression models which included all significantly associated risk factors, descriptive variables including geographical types, gender, race, age, socioeconomic categories and obesity, as well as the dichotomous outcome variable of either hypertension or multimorbidity. Directed Acyclic Graphs (DAG) were used to identify potential confounding variables among the selected variables for both multimorbidity and hypertension. Race and the urban/rural geographies were identified as potential confounding variables for both hypertension and multimorbidity, and were controlled for in both models. Age (p < 0.001) and gender (p < 0.001) had statistically significant associations with both hypertension and multimorbidity as revealed by chi-square tests, and both variables were also controlled for in the final model. The primary exposure variable for both hypertension and multimorbidity was socioeconomic status. Collinearity was tested for but none was found between any variables. Tests for potential interactions were carried out between obesity and age by creating interaction terms. These interaction terms did not contribute significantly to the final model and were not included.

### 2.3 Spatial analysis

The 52 districts of South Africa, as delineated by the 2011 national Census, were the spatial unit of analysis. The district level is the second administrative level of South Africa, below the provinces. Due to the limitations around data availability and a lack of representativeness below a national level, the age-adjusted prevalence of hypertension and multimorbidity were recalculated to represent age-adjusted prevalence for the NIDS study sample within each district. These rates were mapped using Geographic Information Systems (ESRI ArcGIS 10.3 desktop). Socioeconomic disadvantage was mapped and calculated as the percentage of the study sample within each district that were classified as being socioeconomically disadvantaged.

#### 2.3.1 Spatial statistical tools

##### 2.3.1.1 Global index of spatial autocorrelation (Moran’s I)

The Global Moran’s I statistic was used to assess if there was clustering of multimorbidity across the South African districts, or if districts with similar multimorbidity prevalence were spatially situated near each other. The null hypothesis is that multimorbidity is distributed randomly across districts. Moran’s I values generally range between −1 (perfect dispersion) to +1 (perfect clustering) (Legendre and Fortin, 1989). Moran’s I was used in conjunction with G statistics to allow for better insight into local spatial patterns (Getis and Ord, 1992).

##### 2.3.1.2 Getis-Ord Gi* Hot spot analysis

Hot Spot analysis by means of the Getis-Ord Gi* statistic was used to identify statistically significant hot and cold spots of multimorbidity across South African districts. In this study, a hot spot was defined as a clustering of high prevalence rates while a cold spot represented the clustering of low prevalence rates. Regarding conceptualisation of spatial relationships, our study area comprised the 52 districts of South Africa, of which many had only a few neighbouring districts and three were identified as spatial outliers. A spatial weights matrix was applied in the hot spot analysis in order to quantify the spatial relationships of multimorbidity that exist among the districts. A spatial weights matrix was created using Delaunay triangulation, which addresses the challenge of variations in features sizes, as used by Rossen et al. (2014).

## 3 Results

Table 1 displays the descriptive statistics for the NIDS adult sub-sample for wave 1, with adults classified as respondents aged 15 years and older. The study sample for wave 1 comprised 18 526 adults, which included respondents from the Child questionnaire who were 15 years old and older, adults from the Proxy questionnaire and adults who refused to participate in wave 1 but were still part of the panel study and would be interviewed in future waves.

In wave 3 (data not shown), the adult sub-sample for South Africa was 20 015 participants, of which 44.28% were male and 55.72% were female. Proportions of ethnic group (Black African: 77.05%; Coloured: 15.24%; Asian/Indian: 1.72%; White: 6.00%) and geographical types (Rural: 48.28%; Urban: 51.72%) remained similar to those of wave 1.

**Fig. 1:**
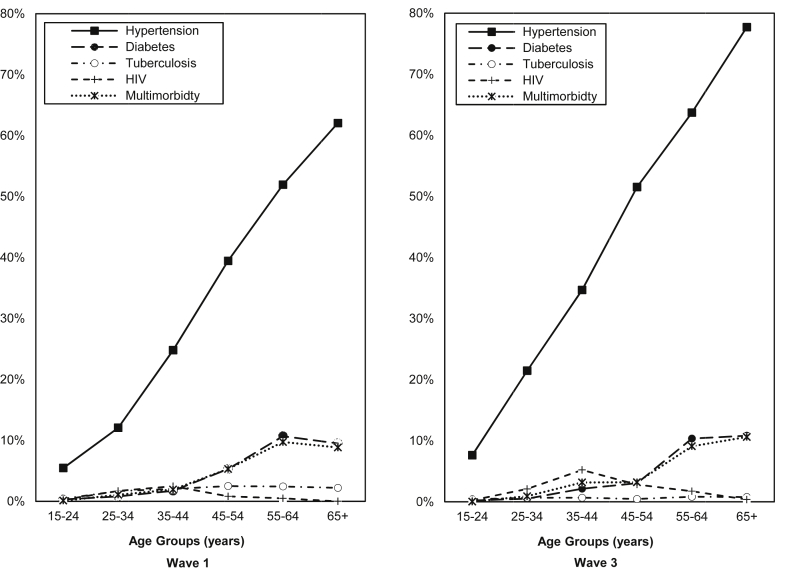
The estimated prevalence of hypertension, diabetes, tuberculosis, human immunodeficiency virus (HIV), and multimorbidity by adult age group for the South African population for wave 1 (2008) and wave 3 (2012) of the National Income Dynamics Study.

Age-adjusted prevalence rates for the South African adult population for waves 1 (2008) and 3 (2012) revealed that hypertension was the most prevalent of the selected diseases (Table 2). In addition, the prevalence rates of health conditions across adult age groups (Fig. 1) showed that hypertension was the most prevalent of the selected health conditions across all ages for both waves. Hypertension was strongly associated with age (p < 0.01) in both waves and there appeared to be a noticeable acceleration in rate of prevalence after the 25–34 age group in wave 1.

In both wave 1 and wave 3, multimorbidity was most prevalent in older adults and had a similar prevalence rate pattern to diabetes across age groups. In wave 1, the decline in multimorbidity prevalence between the 55–64 and 65 + age groups may possibly be linked to the slight decline in prevalence of diabetes, as well as the decrease in TB prevalence rates across these age groups from 2.43% (55–64 years) to 2.21% (65 + years) and the decline of HIV from 0.49% (55–64 years) to 0.00% (65 + years). An increase in prevalence rates for both multimorbidity and diabetes is seen after the 35–44 age group until the 55–64 age group for wave 1.

In wave 3, the noticeably lower multimorbidity prevalence rate for the 45–54 age group may be attributed to the observed lower diabetes rate for the 45–54 age group in wave 3 compared to wave 1, as well as the decline in TB rate for this age group between wave 1 (2.50%) and wave 3 (0.46%).

### 3.1 Temporal analysis of multimorbidity

**Fig. 2:**
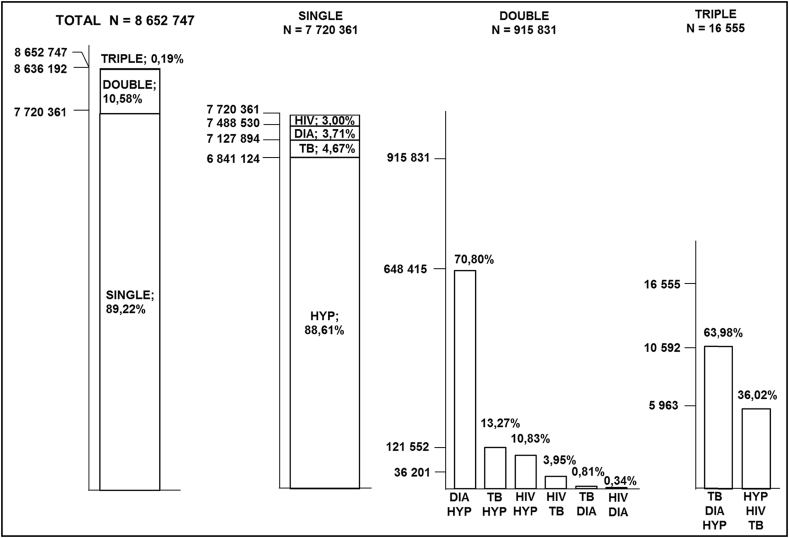
The pattern and distribution of existing single, double and triple disease morbidities in the South African adult population for wave 1 (2008). Key: HIV human immunodeficiency virus; DIA Diabetes; TB tuberculosis; HYP hypertension.

Fig. 2 displays a schematic detailing the single disease morbidities as well as the double (two coexisting diseases) and triple (three coexisting diseases) disease multimorbidities in the South African adult population for wave 1. Hypertension accounted for the majority of single disease prevalence and is shown to coexist most frequently with diabetes (DIA HYP), which together contribute to 70.80% of all double disease multimorbidities. Hypertension is also seen to coexist with both TB and HIV.

**Fig. 3:**
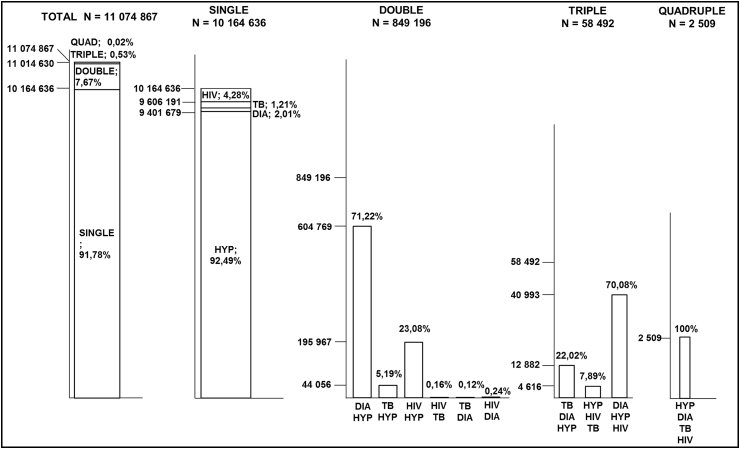
The pattern and distribution of existing single, double and triple disease morbidities in the South African adult population for wave 3 (2012). Key: HIV human immunodeficiency virus; DIA Diabetes; TB tuberculosis; HYP hypertension.

In wave 3 (Fig. 3), hypertension remained the greatest contributor to single disease morbidities (92.49%), while the DIA HYP multimorbidity increased to 71.22% of all double disease multimorbidities. Wave 3 was the only wave with a quadruple burden of multimorbidity (HYP DIA TB HIV), which represented less than 0.01% (N = 2509) of the South African population.

### 3.2 Multivariable analysis results and the association with socioeconomic disadvantage

#### 3.2.1 Hypertension

Results of the multivariable logistic regression of hypertension and associated explanatory variables (see Table 3 for Unadjusted Odds Ratio (OR) and 95% confidence intervals) showed that compared to those who were ‘Not Deprived’, the unadjusted odds of respondents having hypertension were 1.5 times greater for those who were socioeconomically ‘Vulnerable’ (95% CI 1.24–1.87), 1.8 times greater for those socioeconomically ‘Deprived’ (95% CI 1.52–2.22) and 2.1 times greater for those in ‘Severe Poverty’ (95% CI 1.53–2.89). The unadjusted model showed a noticeable association between hypertension and age, where adults older than 65 years were 28.3 times more likely to have hypertension (95% CI 21.18–37.87) relative to baseline 15–24 age group. In addition, respondents who had hypertension were significantly more likely to be female (p < 0.001), obese (p < 0.001), and likely to not exercise (p < 0.001). The unadjusted results showed no significant association between hypertension and rural/urban geographies, smoking status or alcohol status, and only the White racial group was a statistically significant predictor of hypertension relative to the other racial groups.

In the full model, the variables of age and gender were controlled for and the identified confounding variables of race and urban/rural geographies were included. The results showed that respondents living in urban areas were 1.3 times more likely to have hypertension (95% CI 1.05–1.54) compared to respondents in rural areas, which was statistically significant (p < 0.05). Hypertension was also significantly (p < 0.01) associated with obesity (1.93; 95% CI 1.58–2.36). Although exercise was significantly (p < 0.01) associated with hypertension in the bivariate analysis (unadjusted model), it was no longer significant once obesity was included and thus the final model excluded exercise.

In the final model, the categorical variable of race suggested that Coloured respondents (1.37; 95% CI 1.11–1.70) have the highest odds of having hypertension. Respondents who were classified as socioeconomically ‘Vulnerable’ still had the highest OR for hypertension (1.28; 95% CI 1.05–1.55). The age group variable (p < 0.01) was the strongest predictor for hypertension while obesity also contributed significantly to the model (p < 0.01).

#### 3.2.2 Multimorbidity

Logistic regression of multimorbidity and associated variables (see Table 4 for Unadjusted OR and 95% confidence intervals) revealed that the unadjusted odds of respondents having multimorbidity were significant (p < 0.01) and highest for respondents who were socioeconomically ‘Deprived’. These respondents were 1.9 times more likely (95% CI, 1.34–2.72) to have multimorbidity, compared to respondents who were ‘Not Deprived’. Respondents who were classified as ‘Vulnerable’ or in ‘Severe Poverty’ were 1.2 times (95% CI, 0.85–1.67) and 1.1 times (95% CI 0.64–2.00) more likely to have multimorbidity compared to those classified as ‘Not Deprived’, respectively. The odds of having multimorbidity increased with age until the 55–64 age group (9.89; 95% CI 5.36, 18.25) but lowered to 8.9 (95% CI 4.41–17.89) for the 65 + age group, relative to the 25–34 age group. The 15–34 age group was omitted in this model due to an insufficient number of observations. The unadjusted OR showed that respondents who had multimorbidity were likely to be female (1.59; 95% CI 1.23–2.05) and obese (2.18; 95% CI 1.45–3.27). Racial comparisons relative to Black Africans (base group) showed that Asians/Indians were 2.74 times more likely to have multimorbidity (95% CI 1.47–5.14).

In the full model, age was still a strong predictor of multimorbidity, however gender was no longer significantly contributing to the model once obesity was included. These results showed that the odds of having multimorbidity increase with age until the 55–64 age group (8.63; 95% CI 4.47–16.64) and are higher for females (1.17; 95% CI 0.86–1.60), the socioeconomically ‘Deprived’ (1.50; 95% CI 1.00–2.25), Asians/Indians (2.38; 95% CI 1.15, 4.94), respondents living in an urban geography (1.87; 95% CI 1.32–2.66) or respondents who are obese (1.66; 95% CI 1.08–2.54). Whites (0.45; 95% CI 0.21–0.95) had the lowest odds of having multimorbidity. Alcohol, exercise, and smoking variables were omitted as they were not found to be statistically associated with multimorbidity in the bivariate analysis of the unadjusted model.

### 3.3 Spatio-temporal analysis: socioeconomic disadvantage, hypertension and multimorbidity

**Fig. 4:**
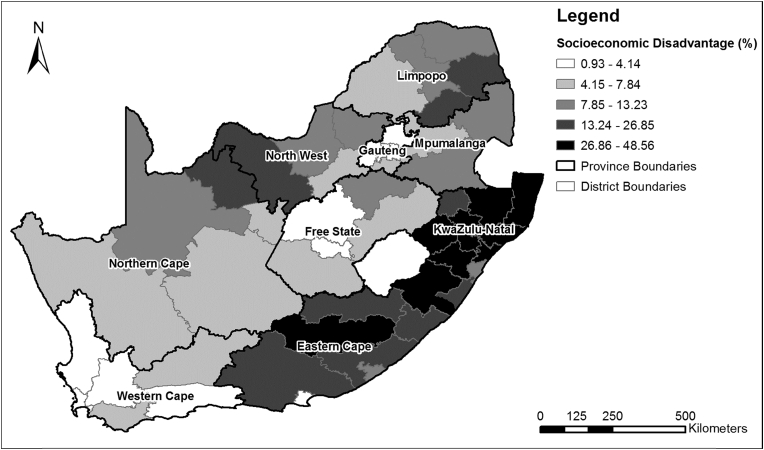
The proportion of the National Income Dynamics Study wave 1 (2008) South African adult sub-sample classified as being socioeconomically disadvantaged (i.e. either deprived or in severe poverty) within each district municipality.

The prevalence of socioeconomic disadvantage was mapped by district (Fig. 4). Of the 52 districts, nine had a socioeconomic disadvantage rate ≥26.86% (i.e. ≥ the highest quantile). These districts were predominately located in the eastern (KwaZulu-Natal Province (KZN)) and south-eastern (Eastern Cape Province) parts of the country. A total of 11 districts had a socioeconomic disadvantage rate ≤4.14% (i.e. ≤ the lowest quantile). These districts were mainly found in the south-western (Western Cape) and central (Gauteng and Free State) parts of the country.

#### 3.3.1 Hypertension

**Fig. 5:**
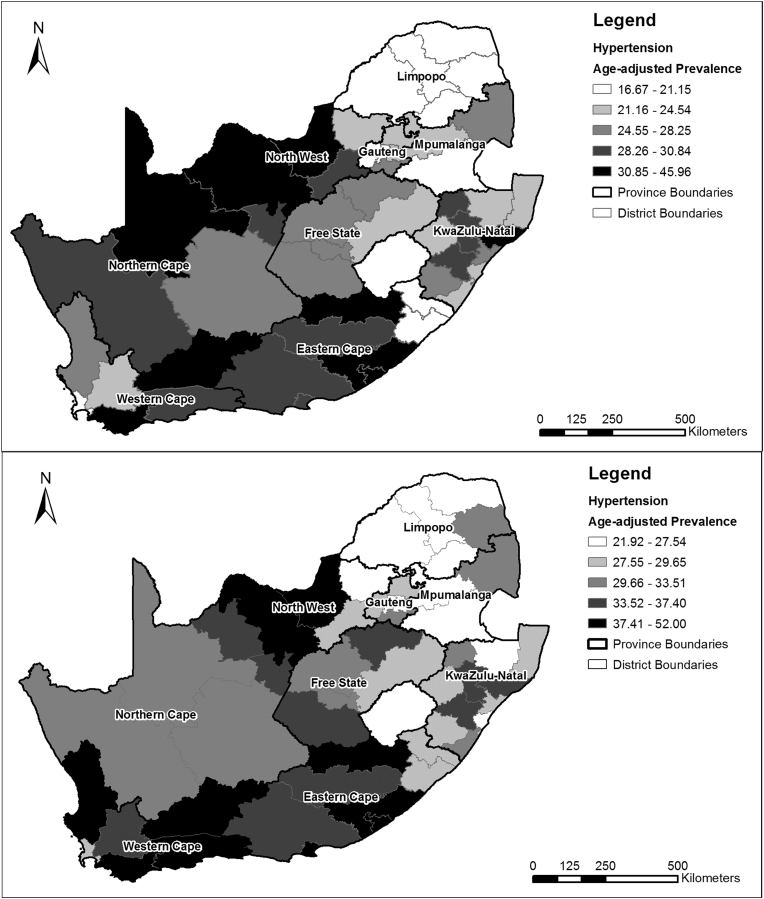
Age-adjusted prevalence of hypertension in the National Income Dynamics Study South African adult sub-sample by district municipality for wave 1 (top) and wave 3 (bottom).

Age-adjusted hypertension prevalence rates for the wave 1 and wave 3 adult sample are shown in Fig. 5 by district. In both waves, lower prevalence rates can be seen in the north of the country (Limpopo and Mpumalanga provinces). In wave 1, higher prevalence rates were predominately in the North West and Northern Cape provinces, as well as in the Eastern Cape. Only one district in KZN had a hypertension prevalence rate ≥30.85% (i.e. ≥ the highest quantile). In wave 3, four of the six districts in the Western Cape had prevalence rates ≥37.41% (i.e ≥ the highest quantile). No district in KZN had a prevalence rate ≥ the highest quantile in wave 3. These patterns do not correspond with the socioeconomic disadvantage spatial pattern.

#### 3.3.2 Multimorbidity

**Fig. 6:**
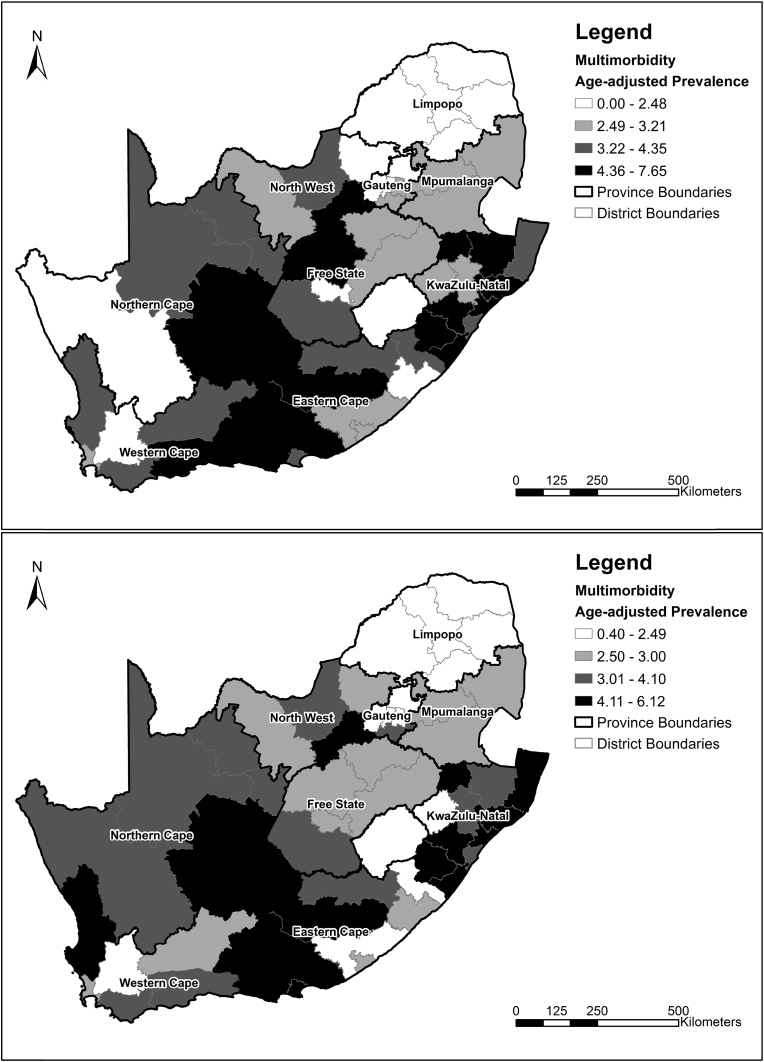
Age-adjusted prevalence of multimorbidity in the National Income Dynamics Study South African adult sub-sample by district municipality for wave 1 (top) and wave 3 (bottom).

The age-adjusted prevalence rates for multimorbidity by district for wave 1 and 3 (Fig. 6) showed lower multimorbidity prevalence rates for Limpopo province, where all five districts had a prevalence rate ≤ the lowest quantile (≤2.48% (wave 1); ≤2.49% (wave 3)). In both waves, seven out of the 11 KwaZulu-Natal districts had prevalence rates ≥ the highest quantile (≥4.36% (wave 1); ≥4.11% (wave 3)) which is similar to the spatial pattern of socioeconomic disadvantage.

#### 3.3.3 Global Moran’s I and hot spot analysis (Getis-Ord Gi*)

**Fig. 7:**
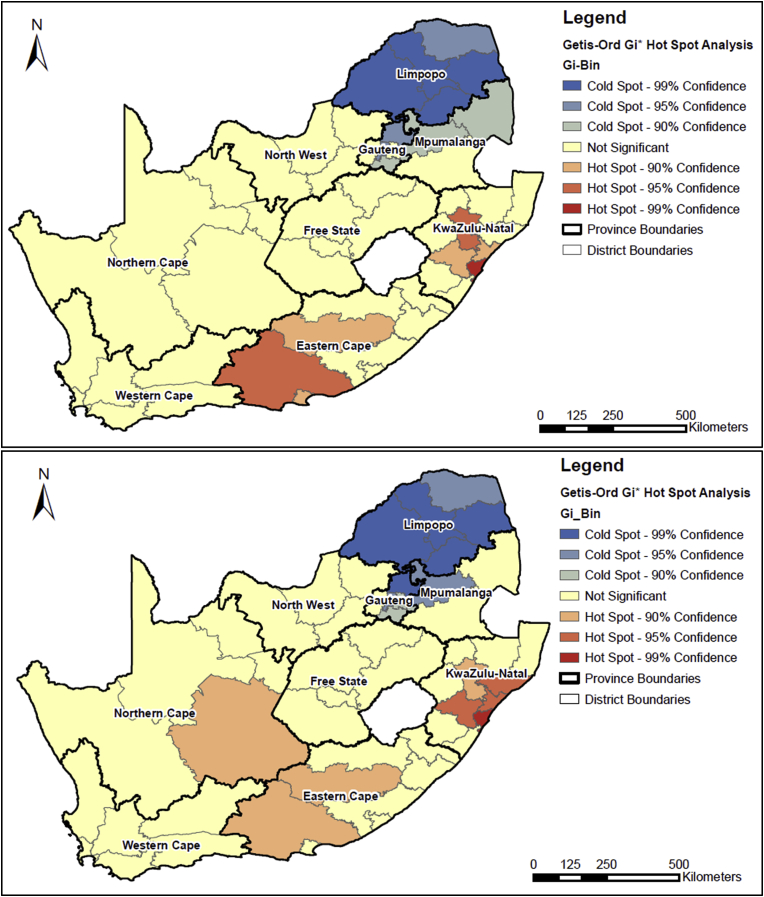
Getis-Ord Gi hot spot analysis of the age-adjusted prevalence rates of multimorbidity in the National Income Dynamics Study South African adult sub-sample by district municipality for wave 1 (top) and wave 3 (bottom).

Assessing the spatial autocorrelation of multimorbidity across districts, the Global Moran’s I test revealed statistically significant (p < 0.001) clustering of multimorbidity (Moran’s I: 0.25; z-score: 3.34). The results of the hot spot (Getis-Ord Gi* statistic) revealed that KwaZulu-Natal and the Eastern Cape do have statistically significant district hot spots in both waves (Fig. 7), which corresponds with the socioeconomic disadvantage spatial pattern. However, one district in the Northern Cape also reveals a hot spot in wave 3. Both waves identified cold spots in Limpopo and Mpumalanga which indicate a statistically significant clustering of lower prevalence of multimorbidity among neighbouring districts that are non-random. However, these cold spots do not appear to correspond to the pattern in the socioeconomic disadvantage map.

## 4 Discussion

This study utilised the NIDS to provide cross-sectional and spatial information on HIV/TB/NCD multimorbidity and to assess the association with socioeconomic disadvantage in South Africa for wave 1 (2008) and wave 3 (2010).

Firstly, the results showed that both infectious and non-communicable diseases are present in South Africa, thereby supporting a protracted-polarised model of epidemiological transition. However, hypertension is a significant health burden in South Africa and continues to grow in prevalence with time. The results for wave 1 were consistent with those of the 1998 Demographic and Health Survey and this study presents an 8.24% increase in hypertension prevalence from 1998 to 2008 (wave 1). Of note, there was a considerable increase of 41.36% in the prevalence of hypertension between 2008 (wave 1) and 2012 (wave 3). A possible reason for the considerable increase in hypertension, compared to that of 1998–2008, is that the NIDS interviews the same sample for each wave. Due to the chronic condition of hypertension, it is expected that the prevalence of hypertension will increase within the same group of people with time, given that it is strongly associated with age, as confirmed in this study and numerous other studies (Kandala, 2014, Liu et al., 2013, Steyn, 2006).

Secondly, although diabetes was not highly prevalent in the South African adult population, diabetes and hypertension were, together, the most predominant form of multimorbidity, which is supported by other studies (Folb et al., 2015, Oni et al., 2015). The presence of multimorbidities within a population has consequences for public health, as patients will require more complex treatment plans and medical care. This study has shown an increase in the prevalence of multimorbidity in South Africa between wave 1 and 3, and that most people with multimorbidity have two coexisting health conditions. In this study, other common combinations of double morbidities included TB/hypertension and HIV/hypertension, which reemphasises the role that hypertension plays in multimorbidity. Although it is clear that hypertension was a common contributor to multimorbidity, the prevalence of multimorbidity was limited by the prevalence of the other health conditions, particularly that of diabetes. This may be a possible reason for the association between multimorbidity and diabetes, as the coexistence of diabetes together with hypertension makes up the majority of all multimorbidities (Fig. 2, Fig. 3) and therefore the rate of morbidity will naturally follow the rate of the less prevalent of these two diseases, namely diabetes. These results cannot be easily compared to those of other studies, as the multimorbidity variable is often defined differently within each study, with the use of different combinations of diseases and measures. For example, a recent South African study estimated the national prevalence of multimorbidity to be 4%, however the study incorporated TB, high blood pressure, diabetes, asthma and cancer as the selected health conditions for analysis (Alaba and Chola, 2013). This highlights the need for a universal definition and measure of multimorbidity in South Africa.

The results of the multivariable analysis showed that the odds of having hypertension are higher for urban residents and respondents who are obese. This provides some support for the notion that a sedentary lifestyle, often linked to the adoption of a ‘Westernised’ urban lifestyle, increases the risk of NCDs. The odds of having hypertension are also higher for those *vulnerable* to socioeconomic disadvantage. However, in the case of multimorbidity, it is those respondents who are *socioeconomically deprived* who have higher odds of having multimorbidity, despite hypertension contributing significantly to multimorbidity cases. This suggests that the element of socioeconomic disadvantage is likely to be a large contributing factor for multimorbidity. These are interesting findings and suggest that poorer groups of people may be experiencing a rise in NCD prevalence.

The multivariable analysis also revealed that obesity was significantly associated with multimorbidity (p < 0.05). The association between females and multimorbidity was found to be driven by obesity.

While Coloured and Asian/Indian respondents had a higher likelihood of having hypertension and multimorbidity, respectively, the association with race is challenging to understand. The legacy of apartheid developed an intrinsic link between race and socioeconomic status, of which the remnants are still visible today. Race could also represent cultural differences existing between ethnic groups that produce variations in risk factors, including diet and behavioural choices such as smoking; however it is also important to acknowledge that some individuals may not strongly identify with their allotted cultural group and thus do not have the stereotypical cultural risk factors (Shim, 2005). Applying Shim’s argument (Shim, 2005), racialization itself, particularly during Apartheid, may have predisposed racial groups to hypertension – a known stress-related disease. These findings, which need to be considered carefully, highlight the need for further investigation into the many social relations and structures that exist inside the association between racial groups and hypertension and multimorbidity in South Africa. Gender has a very similar complexity, in which there are various underlying social structures and processes that are hidden by the simple categorisation of respondents into male and female categories.

The spatial analysis revealed that socioeconomic disadvantage does not appear to reflect the spatial pattern of hypertension but did show similarities to the spatial pattern of multimorbidity, particularly in the Eastern Cape and KwaZulu-Natal provinces, through hot spot analysis. The distribution of higher socioeconomic disadvantage prevalence in Eastern Cape and KwaZulu-Natal provinces is supported by the findings of Noble and Wright (2013), who identified these areas as being predominately rural former homelands, which were previously set aside for Black African ethnic groups during apartheid in 1951, and are characterised by high levels of poverty and deprivation. However, it is likely that the district level may have been too expansive to truly represent the spatial differences in socioeconomic status, multimorbidity and hypertension. These spatial differences are likely to be more discernible at a smaller spatial scale, such as at the urban/rural or intra-urban spatial level.

### 4.1 Strengths and limitations

There are some disadvantages to the use of secondary data. This study was limited to the data available in the NIDS, and thus the variables selected for this study were limited to the quality of the variable data, responsiveness of respondents, as well as the relevance and specificity of the questions in the NIDS survey. In addition, the estimated prevalence of all four diseases relied partially, if not completely, on self-reported data. Whilst TB burden is normally reported as case notification rates, not prevalence, the prevalence of diabetes and HIV have been well described. The estimated diabetes prevalence rate for South African adults (20–79 years) was 6.5% in 2011 (Whiting et al., 2011), while the 2012 HIV prevalence was estimated to be 12.2% (Shisana et al., 2014). These estimates suggest that both diabetes and HIV were underreported in the NIDS. Hypertension is likely to have been underestimated if respondents self-reported a hypertension diagnosis in situations where a healthcare professional identified them as only being pre-hypertensive. However, hypertension prevalence could have been overestimated due to the white coat effect, in which elevated blood pressure may be attributed to visiting a healthcare professional or entering a medical setting (Verdecchia et al., 1995). Language barriers surrounding the diagnosis of diabetes could have resulted in a possible underestimation of prevalence, while stigmas around having HIV and TB may also lead to underreporting. One needs to acknowledge the possibility of potential reverse causation bias in studies that use cross-sectional methods, as people who were aware of their health status would have had the opportunity to change their lifestyle and adopt more healthy habits, such as exercising and losing weight (Liu et al., 2013). Other limitations include the NIDS data restrictions which permit researchers from using results below a district level, such as density calculations and neighbourhood level hot spot analysis, as these would have been useful in interrogating the results of the Getis-Ord Gi* hot spot analysis. Besides, the two-stage sampling design of the survey may introduce uncertainty in the samples, therefore, the hot spots in this study should be interpreted with caution.

Nevertheless, the strengths of this study include the use of a large study sample representative of the country, which includes respondents from all nine provinces and all 52 districts, and uses a combination of both epidemiological and spatial tools to generate new health information for South Africa. Furthermore, this study provides evidence of the burden of hypertension and multimorbidity in South Africa, and has provided a contribution to the conversation around self-reported health data, particularly concerning diabetes, HIV and TB. In addition, the results of this study may be used to inform and promote healthy public policies that support the prevention and control of prevalent diseases and risk factors in the population.

## 5 Conclusion

This study contributes spatial and temporal knowledge of the prevalence of multimorbidity and hypertension in South Africa and has provided a baseline investigation on the association between multimorbidity and socioeconomic disadvantage at the national level. Findings suggest that HIV/TB/NCD multimorbidity is likely to increase in aging populations in South Africa and is largely driven by the relationship between diabetes and hypertension. It is likely that multimorbidity is also driven by a relationship between HIV and TB, yet the nature of self-reported health data makes this difficult to investigate due to stigma around having these health conditions. The finding that multimorbidity is associated with socioeconomic disadvantage (which was measured under the three themes of health, education and living conditions) has implications for government, urban planners and policy makers. However, action at the district level may be futile and further research will be needed to investigate the association between socioeconomic disadvantage and multimorbidity below the district level. In addition, future research should include deeper investigation into spatial complexities of multimorbidity. Mapping multimorbidity suggests possible spatial dependency in residuals of the logistic regression models. Spatial logistic regression modelling (Wu and Zhang, 2013, Paciorek, 2007), may be used to test the clustering of residuals and account for the spatial dependency. Once further waves of the NIDS have been released, survival analysis of key health variables will provide useful insight into multimorbidity. Qualitative research that investigates the possible determinants of multimorbidity, particularly in respondents with hypertension, will greatly contribute to further understanding the burden and drivers of disease in South Africa.

## Figures and Tables

**Table 1:** Unweighted descriptive statistics for the South African adult sub-sample in wave 1 of the National Income Dynamics Study.

Variable	N	Median/percentage	IQR/frequency	Range
Age				
15–24		30.65%	5678	
25–34		19.75%	3659	
35–44		16.63%	3080	
45–54		13.81%	2559	
55–64		9.57%	1773	
65+		9.59%	1777	
Total	18,526	34	22–50	15–105
Gender	18,525			
Male		43.69%	8093	
Female		56.31%	10,432	
Race	18,526			
Black African		76.58%	14,188	
Coloured		15.36%	2845	
Asian/Indian		1.72%	319	
White		6.34%	1174	
Rural/Urban	18,526			
Rural		49.87%	9238	
Urban		50.13%	9288	
Socioeconomic status	18,082			
Not Deprived		66.77%	12,073	
Vulnerable		18.71%	3383	
Deprived		11.90%	2151	
Severe Poverty		2.63%	475	
Alcohol drinking status	15,484			
Never		65.45%	10,134	
Drinker		34.55%	5350	
Smoking status	15,463			
Never		74.42%	11,507	
Smoker		25.58%	3956	
Exercise	15,437			
Never		70.08%	10,818	
Exercise		29.92%	4619	

**Table 2:** Estimated age-adjusted prevalence rates and 95% confidence intervals for the South African adult population for wave 1 (2008) and wave 3 (2012) of the National Income Dynamics Study.

	Wave 1 (age-adjusted)		Wave 3 (age-adjusted)	
Prevalence (%)	95% CI	Prevalence (%)	95% CI
Hypertension	22.73%	(22.13–23.34)	32.13%	(31.48–32.78)
Diabetes	2.81%	(2.58–3.06)	2.71%	(2.49–2.94)
Tuberculosis	1.59%	(1.42–1.78)	0.59%	(0.49–0.71)
HIV	1.11%	(0.97–1.27)	2.13%	(1.93–2.34)
Multimorbidity	2.73%	(2.50–2.98)	2.84%	(2.61–3.08)
TOTAL POP	33,992,872		32,081,996	

**Table 3:** Logistic regression analyses of factors affecting hypertension.

Hypertension	Model 1 – unadjusted		Model 2 – full		Model 3 – final	
OR	(95% CI)	OR	(95% CI)	OR	(95% CI)
Age						
15–24	(base)		(base)		(base)	
25–34	2.38**	(1.90, 2.98)	1.97**	(1.40, 2.76)	1.97**	(1.40, 2.76)
35–44	5.71**	(4.56, 7.15)	4.73**	(3.40, 6.59)	4.74**	(3.40, 6.59)
45–54	11.29**	(8.74, 14.58)	8.93**	(6.28, 12.71)	8.94**	(6.26, 12.78)
55–64	18.73**	(14.05, 24.95)	15.88**	(11.01, 22.89)	15.85**	(10.97, 22.90)
65+	28.32**	(21.18, 37.87)	25.83**	(17.53, 38.05)	25.60**	(17.31, 37.85)
Gender						
Males	(base)		(base)		(base)	
Females	1.61**	(1.41, 1.83)	1.13	(0.95, 1.33)	1.12	(0.96, 1.30)
Socioeconomic status						
Not deprived	(base)		(base)		(base)	
Vulnerable	1.52**	(1.24, 1.87)	1.28**	(1.06, 1.55)	1.28*	(1.05, 1.55)
Deprived	1.84**	(1.52, 2.22)	1.13	(0.89, 1.43)	1.12	(0.89, 1.42)
Severe Poverty	2.10**	(1.53, 2.89)	1.08	(0.79, 1.49)	1.07	(0.78, 1.47)
Race						
Black African	(base)		(base)		(base)	
Coloured	1.23	(1.00, 1.52)	1.37**	(1.1, 1.71)	1.37**	(1.11, 1.70)
Asian/Indian	1.08	(0.66, 1.76)	1.06	(0.57, 1.95)	1.06	(0.57, 1.97)
White	1.30*	(1.02, 1.65)	0.74	(0.50, 1.10)	0.76	(0.51, 1.11)
Rural/Urban						
Rural	(base)		(base)		(base)	
Urban	0.99	(0.86, 1.13)	1.27*	(1.05, 1.54)	1.27*	(1.05, 1.54)
Obesity						
Not obese	(base)		(base)		(base)	
Obese	2.67**	(2.23, 3.19)	1.93**	(1.58, 2.36)	1.93**	(1.58, 2.36)
Exercise						
Exercises	(base)		(base)			
Does not exercise	1.49**	(1.29, 1.73)	0.97	(0.80, 1.16)		
Smoking						
Does not smoke	(base)					
Smokes/smoked regularly	1.11	(0.98, 1.25)				
Alcohol						
Does not drink	(base)					
Drinks alcohol	1.05	(0.93, 1.19)				

*p < 0.05; **p < 0.01.

**Table 4:** Logistic regression analyses of factors affecting multimorbidity.

Multimorbidity	Model 1 – unadjusted		Model 2 – final	
OR	(95% CI)	OR	(95% CI)
Age				
25–34	(base)		(base)	
35–44	1.80*	(1.11, 2.92)	1.64	(0.96, 2.81)
45–54	5.15**	(2.58, 10.29)	4.07**	(1.95, 8.49)
55–64	9.89**	(5.36, 18.25)	8.63**	(4.47, 16.64)
65+	8.89**	(4.41, 17.89)	7.61**	(3.57, 16.20)
Gender				
Males	(base)		(base)	
Females	1.59**	(1.23, 2.05)	1.17	(0.86, 1.60)
Socioeconomic status				
Not deprived	(base)		(base)	
Vulnerable	1.19	(0.85, 1.67)	0.96	(0.65, 1.43)
Deprived	1.91**	(1.34, 2.72)	1.50*	(1.00, 2.25)
Severe Poverty	1.13	(0.64, 2.00)	0.85	(0.45, 1.62)
Race				
Black African	(base)		(base)	
Coloured	1.02	(0.75, 1.41)	0.83	(0.54, 1.27)
Asian/Indian	2.74**	(1.47, 5.14)	2.38*	(1.15, 4.94)
White	0.69	(0.39, 1.22)	0.45*	(0.21, 0.95)
Rural/Urban				
Rural	(base)		(base)	
Urban	1.13	(0.85, 1.50)	1.87**	(1.32, 2.66)
Obesity				
Not obese	(base)		(base)	
Obese	2.18**	(1.45, 3.27)	1.66*	(1.08, 2.54)
Exercise				
Exercises	(base)			
Does not exercise	1.107	(0.74, 1.64)		
Smoking				
Does not smoke	(base)			
Smokes/smoked regularly	0.92	(0.69, 1.22)		
Alcohol				
Does not drink alcohol	(base)			
Drinks alcohol	0.66	(0.47, 0.94)		

*p < 0.05; **p < 0.01.
